# Effect of metabolic control on the *in vitro* proliferation of peripheral blood mononuclear cells in type 1 and type 2 diabetic patients

**DOI:** 10.1590/S1516-31802006000400009

**Published:** 2006-05-04

**Authors:** Maria Cristina Foss-Freitas, Norma Tiraboschi Foss, Eduardo Antonio Donadi, Milton Cesar Foss

**Keywords:** Cell culture, Lymphocyte transformation, Type I diabetes mellitus, Type II diabetes mellitus, Hyperglycemia, Infection, Cultura de células, Ativação linfocítica, Diabetes mellitus tipo I, Diabetes mellitus tipo II, Hiperglicemia, Infecção

## Abstract

**CONTEXT AND OBJECTIVE:**

Diabetes mellitus is a clinical syndrome that frequently leads to the development of chronic complications and high susceptibility to infections. It is probably due to defective immunological defense, which may be related to metabolic control of the disease. The aim of this study was to evaluate the effect of metabolic control on immune-cell behavior in type 1 and type 2 diabetic patients. For this, the *in vitro* proliferation of peripheral blood mononuclear cells (PBMC) was analyzed in patients with inadequate and adequate metabolic control.

**DESIGN AND SETTING:**

Experimental/laboratory study at a university hospital.

**METHODS:**

Eleven type 1 and thirteen type 2 diabetic patients were studied, together with 21 healthy individuals divided in two groups (11/10), who were matched by sex and age with those diabetic patients. PBMC cultures stimulated with concanavalin-A (Con-A) were used to measure ^3^H-thymidine incorporation after 72 hours of cell culturing. For patients with inadequate metabolic control, culturing was performed on the first day of patient hospitalization and again after intensive treatment to achieve adequate control.

**RESULTS:**

The proliferation index for Con-A-stimulated cultures from type 1 diabetic patients was significantly greater than that for cultures from healthy individuals and type 2 diabetic patients, independent of metabolic control. A negative correlation between the proliferation cell index and body mass index and serum C-reactive protein levels was also observed.

**CONCLUSION:**

The increase in the proliferation capacity of type 1 diabetic T lymphocytes was probably not caused by hyperglycemia and/or insulinopenia related to inadequate metabolic control.

## Resumo

**CONTEXTO E OBJETIVO:**

Diabetes *mellitus* é uma síndrome clínica que freqüentemente leva ao desenvolvimento de complicações crônicas, e também, alta susceptibilidade a infecções, provavelmente devido a um defeito na defesa imunológica, que pode ser relacionada ao controle metabólico da doença. Neste estudo, propomos avaliar o efeito do controle metabólico no comportamento imunocelular de pacientes diabéticos tipo 1 e 2 através da proliferação *in vitro* de células mononucleares de sangue periférico (PBMC) em pacientes com controle metabólico inadequado e adequado.

**TIPO DE ESTUDO E LOCAL:**

Estudo experimental e laboratorial, realizado em hospital universitário.

**MÉTODOS:**

Estudamos 11 diabéticos do tipo 1 e 13 do tipo 2 além de 21 controles saudáveis, divididos em dois grupos (11/10), pareados para sexo e idade aos diabéticos tipo 1 e 2. Usamos culturas de PBMCs estimuladas com concanavalina (Con-A) para medir a incorporação de ^3^H-timidina após 72 horas de cultura de células. As culturas foram realizadas no primeiro dia de internação para os pacientes em controle metabólico inadequado, e repetidas após o controle metabólico adequado.

**RESULTADOS:**

O índice de estimulação das culturas estimuladas com Con-A nos diabéticos tipo 1 foi signi ficantemente maior que na cultura de indivíduos saudáveis e diabéticos tipo 2, independentemente do controle metabólico. Além disso, foi observada uma correlação negativa entre o índice de proliferação e o índice de massa corporal e níveis de proteína-C.

**CONCLUSÃO:**

O aumento na capacidade de proliferação de linfócitos de diabéticos tipo 1 não é causado por hiperglicemia e/ou insulinopenia relacionada a controle inadequado.

## INTRODUCTION

Diabetes mellitus is a chronic clinical syndrome caused by insulin deficiency (defects of insulin secretion and/or action) that causes a set of metabolic abnormalities involving the metabolism of glucose, lipids, protein and other substances. It frequently presents chronic microvascular and macro-vascular complications such as retinopathy, nephropathy, coronariopathy, peripheral and cerebral vascular disease, and peripheral neuropathy. The duration and metabolic control of diabetes mellitus seems to be important for the onset and progression of these complications.^1–3^ Besides vascular and neurological complications, a high susceptibility to infection has been described in diabetes mellitus patients,^4–10^ which may be caused by several defects of the immunological defense mechanism.

Impairment of polymorphonuclear leukocyte phagocytosis and reduction in granulocyte phagocytic capacity have been reported with increased plasma glucose concentration in diabetic patients, and these abnormalities are reversed after insulin therapy.^11–16^ The most dramatic defect that occurs in diabetes mellitus is related to abnormalities of T cell function.^17,18^ The reason for these alterations in the immune cell behavior of diabetic patients is still undefined, with few studies of lymphocyte proliferation and none regarding the influence of metabolic control in diabetic patients.

## OBJECTIVE

Considering the importance of the immune system in the development of diabetic complications such as higher susceptibility to infections in diabetic patients, we proposed to study the *in vitro* proliferation of peripheral blood mononuclear cells (PBMCs) from type 1 and type 2 diabetic patients, and to correlate this with inadequate and adequate metabolic control, as measured by the glycemic para meters associated with body mass index (BMI), and with the peripheral inflammatory response assessed by C-reactive protein levels.

## METHODS

Twenty-four diabetic patients were selected at the outpatient clinics of the university hospital of Faculdade de Medicina de Ribeirão Preto, Universidade de São Paulo (Table 1). We also studied 21 healthy individuals who were matched by sex, age and BMI with these type 1 and type 2 diabetic patients.

**Table 1.** Clinical characteristics of type 1 and type 2 diabetic patients and healthy individuals who were matched with these type 1 (Control 1) and type 2 (Control 2) patients. Data are presented as mean ± standard deviation (SD)

	Type 1 diabetic patients (n = 11)	Type 2 diabetic patients (n = 13)	Control 1 (n = 11)	Control 2 (n = 10)
**Age (years)**	22.7 ± 7.1	49.7 ± 18.2	24.5 ± 7.1	39.5 ± 15.8
**Sex (F/M)**	6/5	3/10	8/3	2/8
**BMI (kg/m^2^)**	23.1 ± 3.8	28.5 ± 8	21.4 ± 2.8	24.7 ± 9.1
**Hbg (%)**	11.7 ± 2	12 ± 2.3	5.7 ± 0.9	5.5 ± 1.6
**DM duration (years)**	9.6 ± 5.8	10.2 ± 7	-	-
**CRP (mg/dl)**	0.2 ± 0.2	0.6 ± 0.5	-	-

*BMI = body mass index; Hbg = glycated hemoglobin; DM = diabetes mellitus; CRP = C-reactive protein. F = female; M = male.*

The patients presented inadequate metabolic control (defined as fasting glucose greater than 200 mg/dl and glycated hemoglobin greater than 11%), but did not present any infectious disease and were not using any drugs that might have interfered with the results. The patients were hospitalized for 2-3 weeks to obtain adequate metabolic control. This was achieved by using an intensive protocol with capillary blood glucose measurements at 7, 11, 17 and 23 hours, and administration of regular insulin injections until a reduction of at least 100 mg/dl in mean daily glycemic level was reached. In addition to the neurological examination, the presence of microvascular complications was evaluated by ophthalmological examination and urine protein measurement, and the presence of macrovascular complications by clinical and electrocardiographic evaluation.

The protocol was approved by the Ethics Committee of Hospital das Clínicas, Faculdade de Medicina de Ribeirão Preto, Universidade de São Paulo (HC-FMRPUSP) and, before giving their consent to participate, the volunteers were carefully informed about the nature, purpose and possible risks of the study.

Blood samples were obtained on the first and last day of hospitalization. PBMCs were isolated by gradient density using Ficoll-Hypaque^®^.^19^ PBMCs at the concentration of 2.5 x 10^6^ cells/ml were cultivated in triplicate in the presence or absence of concanavalin-A (Con-A). After 72 hours of culturing at 37°C in a humid environment with approximately 5% CO_2_, 0.5 microcuries of ^3^H-thymidine were added to each well. The cells were maintained in the same condition as described above for an additional period of 16 hours. They were then collected using an automated PHD cell harvester (Cambridge Technology Inc., Cambridge, UK) and radioactivity was measured over a period of 10 minutes using a scintillation spectrometer (Beckman Coulter Inc., Fullerton, California, USA).

The lymphoproliferation results were evaluated in accordance with the cell incorporation of ^3^H-thymidine. These results are presented as the proliferation index, obtained as the ratio between the mean counts per minute (CPM) from the triplicates of the stimulated culture (Con-A) and non-stimulated culture (culture medium-RPMI). A high-sensitivity assay for C-reactive protein measurement in blood samples was carried out on day 1 using a turbidimetric immunoassay (Dimension^â^, Dade Behring Inc. Newark, USA).

The results are presented as median (M), mean (X) and standard deviation (SD). The GraphPad Prism program (San Diego, California, USA) was used for the statistical analysis. The Kruskal-Wallis test was used to analyze differences between the three groups (type 1 and type 2 patients, and controls), and the Wilcoxon test was used to compare the diabetic groups before and after metabolic control. The Spearman test was used to calculate all correlations. p values < 0.05 were considered to be significant.

## RESULTS

**Figure 1:**
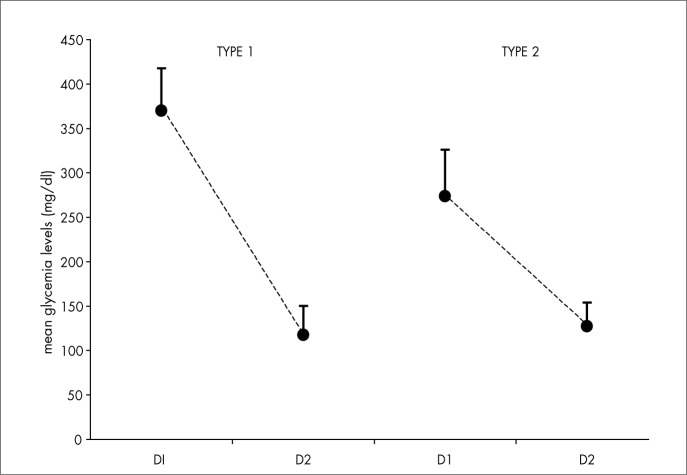
Daily mean glycemic levels of type 1 and type 2 diabetic patients on the first (D1) and last (D2) day of hospitalization.

The three groups studied (type 1 and type 2 diabetic patients and healthy individuals) were statistically similar with regard to age, sex and BMI (Table 1). The patients did not present chronic macrovascular, microvascular or neurological complications. The metabolic control evaluated by daily mean glycemic profiles during the hospitalization period showed a significant decrease with treatment, between the first and last day of hospitalization for type 1 and type 2 diabetic patients (p = 0.0002 and p = 0.0001, respectively, Figure 1).

**Figure 2:**
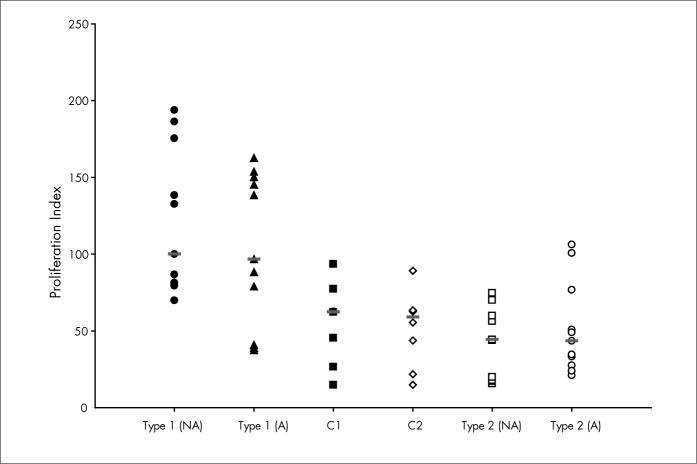
Proliferation index in Con-A (10 μg/ml)-stimulated peripheral blood mono-nuclear cells cultures from type 1 and type 2 diabetic patients with inadequate (NA) and adequate (A) metabolic control, and from healthy individuals who were matched with these type 1 (C1) and type 2 (C2) diabetic patients. The horizontal lines represent the medians for each group.

The proliferation index was higher in the type 1 diabetes group, independent of glycemic control [M = 100.1 (inadequate) and M = 96.8 (adequate)], than in the type 2 group [M = 44.4 (inadequate), p < 0.0001; and M = 43.4 (adequate), p = 0.01] (Figure 2). Type 1 diabetic patients also showed higher proliferation index than for the healthy individuals (M = 62.5, p = 0.01) who were matched for sex and age (Figure 2).

The proliferation index showed a negative correlation (r = − 0.5, p = 0.01) with the BMI of the diabetic patients with both adequate and inadequate metabolic control. A negative correlation (r = − 0.7, p = 0.002) was also observed with the C-reactive protein levels of inadequately controlled diabetic patients.

## DISCUSSION

Lymphoproliferation reflects the capacity of a cell to produce clones *in vitro* in the presence or absence of polyclonal activators. In the present study, concanavalin-A (ConA) was used as a polyclonal T cell activator. The use of soluble polyclonal activators is based on the knowledge that they present superficial epitopes that are recognized by human cell surface receptors, regardless of the presence of illness.

The present study evaluated the lymphoproliferative response and its correlation with glycemic control in diabetic patients, for the first time. Type 1 diabetic lymphocytes showed a higher *in vitro* proliferation rate, independent of glycemic control, which could be explained by some metabolic defects of these diabetic patients. Some authors have suggested that hyperglycemia has a negative influence on immune-competent cells. According to Reinhold et al.,^20^ the amount of thymidine incorporation in PBMC cultures from normal individuals is inversely proportional to the glucose concentration of the culture. This hypothesis is supported by the evidence of improvement in the immune-cell response of diabetic patients following metabolic control.^21^ On the other hand, the insulinopenic state may be the reason for these immunological defects.^22^

Current studies^23,24^ suggest that insulin has an anti-inflammatory effect, even in situations of acute compensation for hyperglycemic metabolic disturbances. In our study, the glycemic control did not determine differences in the proliferation of type 1 diabetic lymphocytes. Hyperglycemia was completely normalized and adequate control was obtained using insulin therapy, making it difficult to conclude whether hyperglycemia and plasma insulin levels are modulators of the lymphocyte response.

Furthermore, the increase in proliferation index for type 1 diabetic patients was significant when compared with type 2 diabetic patients and normal individuals. These results suggest that the increased proliferation index in type 1 diabetic patients is intrinsic to this disease and may be due to chronic activation of the immunological system, thus enhancing the stimulated PBMC response. This chronic lymphocyte activation may be determined by superantigen infections^25,26^ and/or be related to immunological alterations determined by the type 1 autoimmune process. According to McCormack et al.,^27^ chronic exposure to superantigens initially causes a reduction in the number of T cells, probably due to superantigen stimulation that determines proliferation followed by an increased cell death rate.^28,29^ In fact, Gonzalo et al.^30^ showed DNA fragmentation characteristic of apoptosis in T cells after 72 hours of culturing in the presence of superantigens.

However, although we did not observe any influence of metabolic control on cell proliferation, we noticed a negative correlation with BMI and also observed that the highest C-reactive protein levels correlated with the lowest lymphoproliferation values in diabetic patients with inadequate metabolic control. These are important results if we consider that C-reactive protein is an acute inflamma-tory reactive protein that is usually related to degenerative processes induced by pro-inflammatory cytokines like interleucin-6 (IL-6), tumor necrosis factor-α (TNF-α) and others. It may be proposed that, under conditions of inadequate metabolic control, the production of this protein and other pro-inflammatory factors can regulate the immunological system to favor susceptibility to infection.

## CONCLUSIONS

The results obtained in this study show that type 1 diabetic patients have an increased *in vitro* cloning capacity that probably is not due to hyperglycemia and/or insulinopenia.
